# Evaluation of Serum GPR-120 Levels in Diabetic Patients With and Without Nephropathy: A Comparative Study on Lipid and Renal Parameters

**DOI:** 10.7759/cureus.85749

**Published:** 2025-06-11

**Authors:** Divya Singh, Anand Shaker, Tejas D Patel, Mahammed Kaif Bhaniya, Ashishkumar M Agravatt

**Affiliations:** 1 Biochemistry, Parul Institute of Medical Sciences and Research, Parul University, Vadodara, IND; 2 Biochemistry, Swaminarayan Institute of Medical Sciences And Research, Swaminarayan University, Kalol, IND; 3 Medicine, Dr. Kiran C Patel Medical College and Research Institute, Bharuch, IND; 4 Biochemistry, Pandit Dindayal Upadhyay (PDU) Medical College, Rajkot, IND

**Keywords:** chronic kidney disease, cystatin c, diabetic nephropathy, gpr-120, lipid metabolism, microalbuminuria

## Abstract

Background: Diabetic nephropathy (DN) remains one of the main reasons for end-stage renal disease globally and is strongly related to adverse lipid profiles, oxidative stress, and chronic inflammation. The G-protein coupled receptor 120 (GPR-120), a protein implicated in lipid metabolism and anti‐inflammatory pathways, has garnered recent attention as a possible diagnostic and prognostic biomarker reflecting both metabolic derangements and renal injury. However, its clinical importance in diabetic cohorts, particularly those with nephropathy, remains underexplored.

Methods: We performed a case-control study of 200 subjects (adults) with type 2 diabetes mellitus, subdivided into those without nephropathy (n=100) and those with nephropathy (n=100). All subjects underwent comprehensive biochemical profiling, such as fasting glucose, postprandial glucose, glycated hemoglobin (HbA1C), lipid indices (total cholesterol (TC), high-density lipoprotein cholesterol (HDL‐C), low-density lipoprotein cholesterol (LDL‐C), triglycerides, and oxidized LDL), renal characteristic checks (creatinine, cystatin C, microalbuminuria, estimated glomerular filtration rate (eGFR)), and serum GPR‐120 analyzed via enzyme‐linked immunosorbent assay. We compared GPR-120 levels across both groups and performed regression analysis to investigate how GPR-120 correlates with lipid parameters and renal markers.

Results: Subjects with nephropathy exhibited drastically lower GPR‐120 concentrations (2.9 ± 0.8 ng/mL) compared to the ones without nephropathy (11.2 ± 4.5 ng/mL, p<0.001). Regression analysis revealed that HDL‐C positively correlated with GPR‐120 (p<0.001), while LDL‐C, triglycerides, and cystatin C were negatively associated with GPR‐120 (p<0.01). Microalbuminuria additionally showed an inverse relationship with GPR‐120 levels. Considerably higher eGFR values predicted better GPR‐120 concentrations, suggesting a defensive or compensatory mechanism.

Conclusion: Our findings indicate that GPR‐120 is markedly reduced in DN and demonstrates strong associations with both adverse lipid profiles and renal dysfunction. The GPR‐120 may represent an emerging biomarker of integrated metabolic and renal health in diabetes. Larger longitudinal studies are recommended to confirm its prognostic utility and clarify whether interventions targeting dyslipidemia could modulate GPR‐120 levels.

## Introduction

Diabetic nephropathy (DN) remains a significant public health concern, representing the primary contributor to end-stage renal disease globally and imposing enormous economic burdens on healthcare systems [1]. Prolonged hyperglycemia in diabetes triggers complex pathophysiological pathways, including increased polyol pathway flux, accumulation of advanced glycation end products, and oxidative stress, ultimately impacting renal microvasculature [2,3]. In parallel, dyslipidemia with elevated low‐density lipoprotein cholesterol (LDL‐C), triglycerides, and reduced high‐density lipoprotein cholesterol (HDL‐C) exacerbates renal inflammation and endothelial dysfunction [4]. Although microalbuminuria and elevated serum creatinine remain standard clinical predictors of DN, there is growing interest in novel biomarkers that may provide earlier or more nuanced insights into disease progression [5,6,7,8].

The G protein-coupled receptor 120 (GPR120), expressed in adipocytes, macrophages, podocytes, and enteroendocrine cells, has recently gained attention as a possible 'multisystem' modulator, influencing both lipid metabolism and inflammatory signaling [9]. The GPR120, a receptor involved in lipid sensing and anti-inflammatory signaling, may serve as a mechanistic link between dyslipidemia and renal injury in diabetes. Therefore, assessing its relationship with established lipid and renal biomarkers may reveal its potential utility as a marker of metabolic-renal integration [10-12]. This multi-pronged activity suggests that dysregulated GPR-120 might reflect or potentiate detrimental metabolic states, particularly in the context of chronic hyperglycemia, where inflammatory pathways are persistently activated [13]. Despite these suggestive mechanisms, systematic clinical evaluations of serum GPR‐120 levels in DN remain limited, leaving critical questions about its association with key markers, such as cystatin C, estimated glomerular filtration rate (eGFR), and microalbuminuria, unanswered.

Moreover, lipid derangements have consistently been related to the development of DM and the development of DN. Elevated LDL‐C and triglycerides promote the formation of oxidized lipoproteins, aggravating oxidative stress in the glomerular and tubular compartments [14,15]. Simultaneously, reduced HDL‐C attenuates the kidney’s capacity to mobilize and reverse‐transport cholesterol. By evaluating how GPR‐120 parallels or diverges from these known dyslipidemic and nephrotoxic parameters, clinicians and researchers may uncover whether GPR‐120 contributes additional prognostic or mechanistic value beyond traditional markers. This possibility is particularly attractive given ongoing efforts to refine precision medicine approaches for metabolic diseases [16-18].

The primary objective of this study is to compare serum GPR120 levels between diabetic patients with and without nephropathy. The secondary objective is to evaluate correlations between GPR120 levels and lipid (total cholesterol, HDL-C, LDL-C, triglycerides, oxidized LDL) and renal biomarkers (serum creatinine, cystatin C, microalbuminuria, and eGFR). We aimed to assess the potential of serum GPR-120 as a clinically useful biomarker to complement existing diagnostic tools for DN.

## Materials and methods

This retrospective case-control study was conducted at Dr. Kiran C Patel Medical College and Research Institute (Bharuch, GJ, IND), with data collected from patient records between August 2023 to December 2024. A total of 200 adult patients with type 2 diabetes mellitus who were 18 years of age or older were identified for the study. To ensure the reliability and clinical relevance of the findings, participants were selected based on well-defined inclusion and exclusion criteria.

Group 1 featured 100 diabetic patients without nephropathy (eGFR ≥90 mL/min/1.73 m², microalbuminuria <30 mg/day) and diabetics without renal complications. Group 2 comprised 100 diabetic patients with nephropathy, defined by at least two of the following: microalbuminuria >30 mg/day, eGFR <60 mL/min/1.73 m², or elevated cystatin C or creatinine, corresponding to CKD stages 2-4. Patients were excluded if they had type 1 diabetes, active or recent infections, autoimmune or inflammatory disorders, were pregnant women, had gestational diabetes, had incomplete laboratory records, or were enrolled in investigational drug trials. All other potential etiologies of chronic kidney disease (CKD) apart from DN, such as polycystic kidney disease, HIV-associated nephropathy, toxic nephropathy, lupus nephritis, IgA nephropathy, focal segmental glomerulosclerosis, and other autoimmune disorders, were systematically eliminated through clinical evaluation, laboratory testing, and relevant imaging as appropriate. End-stage renal disease (ESRD) patients on dialysis were also excluded to avoid confounding effects on biochemical parameters, including GPR120, such as fluid shifts or altered protein clearance. Non-diabetic renal failure patients were not studied to maintain focus on DN. The sample size was determined based on a power analysis conducted prior to the study, which indicated that a minimum of 200 subjects would provide adequate statistical power to detect significant differences in GPR-120 levels and other parameters between the two groups.

The study protocol was approved by the Institutional Ethics Committee of Dr. Kiran C Patel Medical College and Research Institute (Bharuch, GJ, IND) (approval no. 04/2023) per the Declaration of Helsinki. Data were gathered from pre-existing patient records, and blood samples were simultaneously examined in a clinical biochemistry lab. The design made it easier to use regression models to analyze multiple variables and determine how GPR-120 and lipid profiles relate to one another.

Every patient received routine clinical examinations. Blood samples were collected, centrifuged at 3000 rpm for 10 minutes, and serum was stored at −20°C until analysis. An automated chemistry analyzer was used to measure the levels of glucose during fasting and after meals, and high-performance liquid chromatography (HPLC) was used to determine the glycated hemoglobin (HbA1c). Using common enzymatic techniques, lipid profiles were determined by measuring total cholesterol, HDL-C, LDL-C (when applicable), and triglycerides. Using an enzyme-linked immunosorbent assay (ELISA) kit, the amount of oxidized LDL was measured. The 24-hour urine microalbuminuria, eGFR (modification of diet in renal disease (MDRD) formula), serum creatinine (Jaffe's method), and cystatin C (turbidimetric immunoassay) were used to assess renal function. All samples were processed per manufacturer instructions, and serum GPR-120 levels were determined using a sandwich ELISA kit (FineTest (Fine Biotech, Wuhan, CHN), catalog no. EH3167, detection range: 0.625-40 ng/mL, sensitivity: 0.375 ng/mL) per manufacturer instructions. 

The GPR120 ELISA assay had intra-assay and inter-assay coefficients of variation (CVs) of <6% and <5%, respectively. Patients with incomplete laboratory records were excluded during data extraction to minimize missing data. Hence, no imputation techniques were applied. Statistical analyses were performed using SPSS Statistics version 26.0 (IBM Corp., Armonk, NY, USA). Using suitable statistical tests such as t-tests, continuous variables between groups were compared. Data normality was assessed using the Shapiro-Wilk test, and independent t-tests assumed equal variances unless Levene’s test indicated otherwise. The association between GPR-120 levels and different lipid and renal parameters was investigated using regression analysis. A statistically significant p-value was defined as less than 0.05. 

## Results

**Figure 1:**
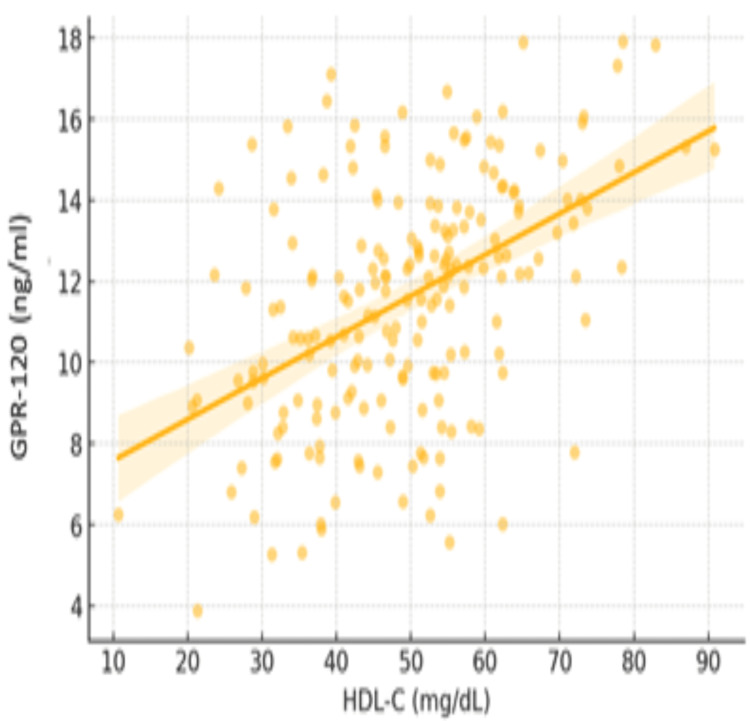
Comparison of the levels of GPR-120 with HDL-C in diabetic patients GPR-120: G-protein coupled receptor 120, HDL-C: High-density lipoprotein cholesterol

A total of 200 patients with type 2 diabetes were enrolled, 100 with DN and 100 without (controls). Demographic, glycemic, and renal characteristics of the study population are summarized in Table 1. Patients with nephropathy were significantly older than those without, though both groups had comparable gender distributions. Glycemic control markers, including fasting blood sugar (FBS) and postprandial blood sugar (PPBS), were notably worse among nephropathy patients. Although HbA1c was higher in the nephropathy group, the difference was not statistically significant (p = 0.059). Renal parameters showed significantly lower eGFR and markedly higher microalbuminuria in the nephropathy. A strong positive correlation was observed between HDL-C and GPR-120 levels (Figure 1). Higher HDL-C levels are associated with increased GPR-120, suggesting that HDL-C might preserve or enhance GPR-120 function, potentially mitigating inflammation and metabolic disturbances in the DN patient group (p < 0.001 for both), highlighting substantial renal dysfunction.

**Table 1:** Baseline demographic, glycemic, and renal parameters in type 2 diabetic patients with and without nephropathy *Statistically significant; FBS: Fasting blood sugar, PPBS: Postprandial blood sugar, HbA1C: Glycated hemoglobin, eGFR: Estimated glomerular filtration rate

Parameter	Non-nephropathy (n=100)	Nephropathy (n=100)	p-value
Age (years)	49.3 ± 8.9	59.4 ± 10.2	<0.001*
Male (%)	52	55	0.632
FBS (mg/dL)	90.7 ± 10.4	175.3 ± 18.8	<0.001*
PPBS (mg/dL)	127.6 ± 21.2	252.1 ± 39.4	<0.001*
HbA1C (%)	6.9 ± 1.2	8.04 ± 1.6	0.059
eGFR (mL/min/1.73 m²)	95.9 ± 11.1	43.2 ± 10.5	<0.001*
Microalbumin (mg/day)	12.6 ± 6.1	421.8 ± 103.4	<0.001*

Table 2 presents a comparative analysis of lipid parameters and GPR-120 levels between the nephropathy and non-nephropathy groups. Patients with nephropathy exhibited significantly elevated total cholesterol, LDL-C, triglycerides, and oxidized LDL, along with markedly reduced HDL-C levels. The GPR-120 concentrations were significantly lower in the nephropathy group, underscoring its potential role in lipid metabolism and renal impairment in diabetic individuals.

**Table 2:** Comparison of lipid profile and GPR-120 levels between diabetic patients with and without nephropathy *Statistically significant; GPR-120: G-protein coupled receptor 120, HDL-C: High-density lipoprotein cholesterol, LDL-C: Low-density lipoprotein cholesterol, LDL: Low-density lipoprotein

Parameter	Non-nephropathy (n=100)	Nephropathy (n=100)	p-value
Total cholesterol (mg/dL)	172.6 ± 32.1	199.6 ± 37.8	<0.001*
HDL-C (mg/dL)	60.2 ± 10.3	29.7 ± 5.2	<0.001*
LDL-C (mg/dL)	106.7 ± 25.4	156.9 ± 42.5	<0.001*
Triglycerides (mg/dL)	128.4 ± 24.8	254.3 ± 56.9	<0.001*
Oxidized LDL (ng/mL)	64.4 ± 12.1	146.9 ± 80.3	0.002*
GPR-120 (ng/mL)	11.2 ± 4.5	2.9 ± 0.8	<0.001*

Regression analysis was performed to explore the predictors of GPR-120 levels. As shown in Table 3, HDL-C and eGFR emerged as significant positive predictors, while LDL-C, triglycerides, microalbumin, and cystatin C were significantly associated with lower GPR-120 concentrations. These findings reinforce the strong interplay between lipid and renal health in modulating GPR-120 expression.

**Table 3:** Regression analysis identifying predictors of outcome and their associations with GPR-120 GPR-120: G-protein coupled receptor 120, HDL-C: High-density lipoprotein cholesterol, LDL-C: Low-density lipoprotein cholesterol, eGFR: Estimated glomerular filtration rate, Unstd.: Unstandardized

Predictor	B (Unstd.)	p-value
HDL-C	+0.10	<0.001
LDL-C	−0.01	0.009
Triglycerides	−0.02	0.012
eGFR	+0.08	<0.001
Microalbumin	−0.03	0.015
Cystatin C	−0.86	0.030

**Figure 2:**
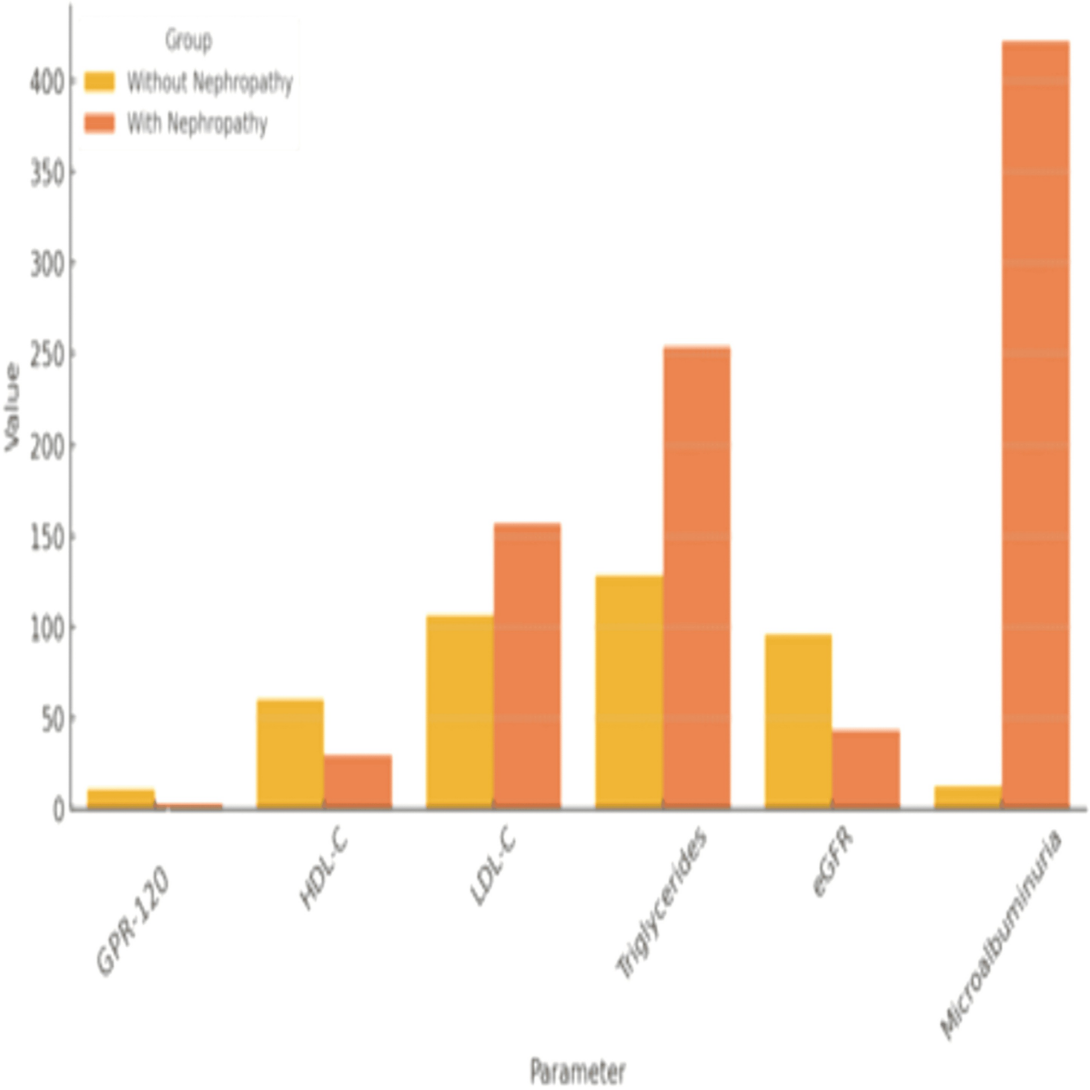
Comparative analysis of GPR-120, lipid, and renal markers in diabetic patients with and without nephropathy GPR-120: G-protein coupled receptor 120, HDL-C: High-density lipoprotein cholesterol, LDL-C: Low-density lipoprotein cholesterol, eGFR: Estimated glomerular filtration rate

Figure 2 compares the GPR-120, lipid, and renal markers across study groups. Patients with nephropathy exhibited significantly lower GPR-120 levels and more adverse lipid and renal profiles compared to those without nephropathy.

**Figure 3:**
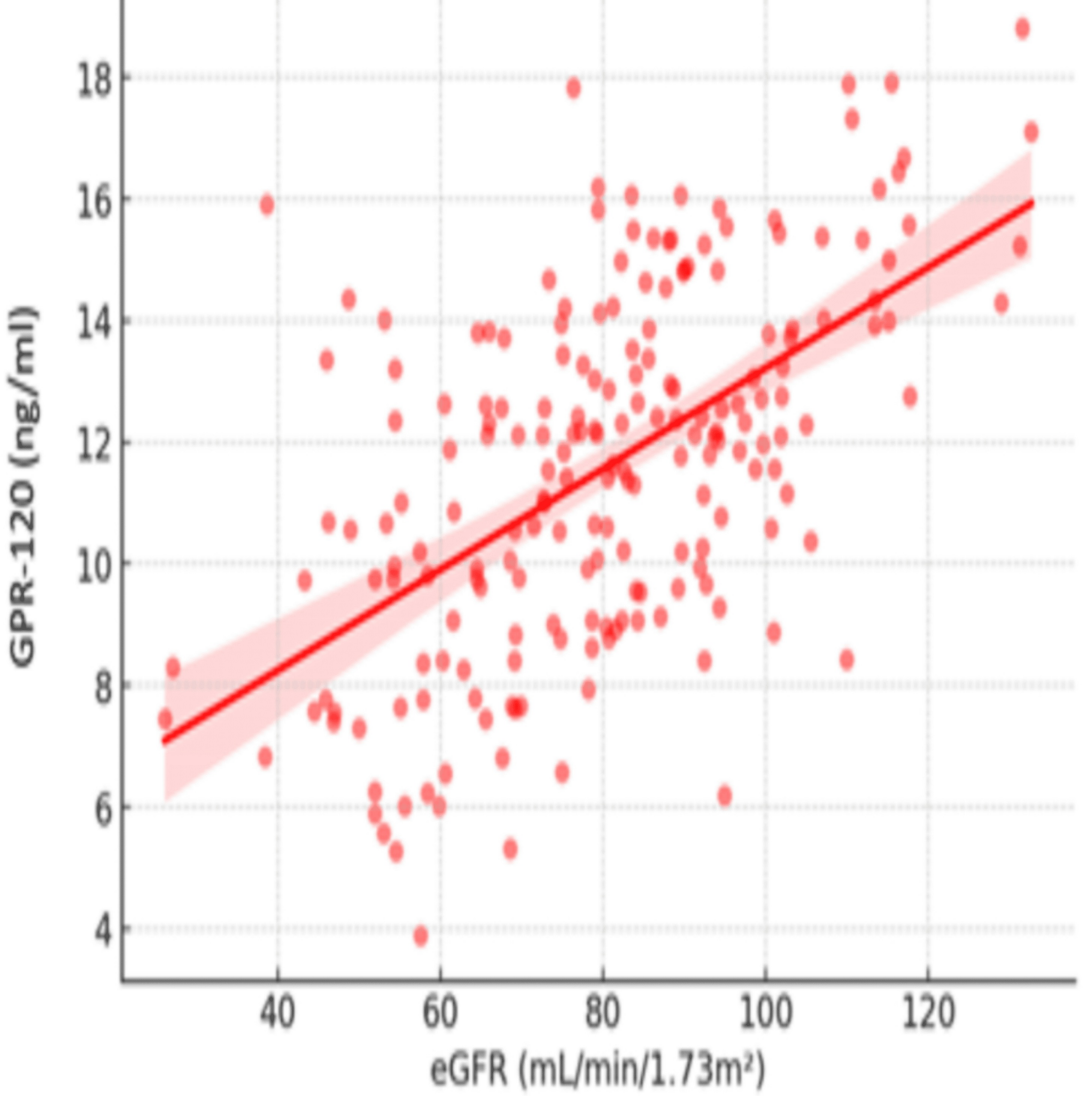
Correlation between GPR-120 and eGFR GPR-120: G-protein coupled receptor 120, eGFR: Estimated glomerular filtration rate

A significant positive correlation between eGFR and GPR-120 levels is shown in Figure 3, suggesting GPR-120's potential as a renal biomarker. As kidney function deteriorates (lower eGFR), GPR-120 concentrations decline, indicating a potential biomarker role for GPR-120 in predicting the progression of DN and associated metabolic-renal dysfunction in high-risk individuals.

**Figure 4:**
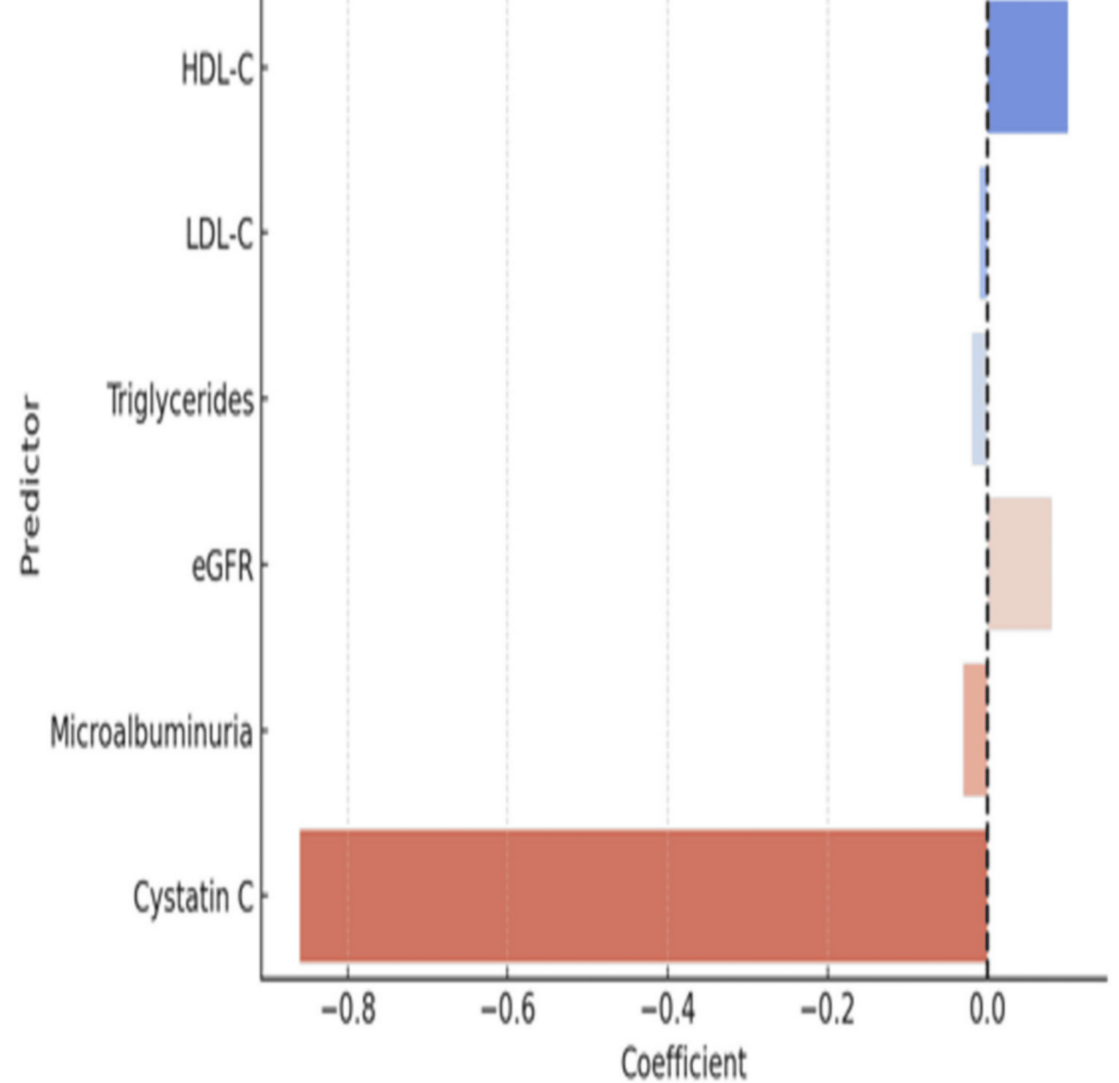
Regression coefficients of GPR-120 with lipid and renal markers GPR-120: G-protein coupled receptor 120, HDL-C: High-density lipoprotein cholesterol, LDL-C: Low-density lipoprotein cholesterol, eGFR: Estimated glomerular filtration rate

Figure 4 presents a summary plot of regression coefficients, illustrating the directional relationships between GPR-120 and key lipid and renal markers. The GPR-120 was positively associated with HDL-C and eGFR, and negatively associated with LDL-C, triglycerides, microalbumin, and cystatin C, reflecting its possible involvement in lipid and renal metabolic regulation.

## Discussion

Our findings demonstrate a robust relationship between decreased GPR‐120 levels and the presence of DN, thus reinforcing the notion that GPR‐120 might be a clinically meaningful biomarker for evaluating metabolic and renal risks among individuals with type 2 diabetes. The dramatically lower GPR‐120 concentrations observed in nephropathy align with the concept that persistent hyperglycemia, oxidative stress, and inflammatory mediators collectively impair the beneficial effects of GPR‐120 in modulating lipid metabolism and inflammatory signaling [19,20]. Notably, GPR-120 activation is known to suppress nuclear factor kappa-light-chain-enhancer of activated B cells (NF-κB)-mediated pro-inflammatory signaling and modulate lipid metabolism by increasing HDL-C and decreasing LDL-C and triglyceride levels. These alterations are likely downstream effects of improved insulin sensitivity and reduced inflammation, rather than parallel events. Furthermore, the strong inverse associations we identified between GPR‐120 and established renal biomarkers, namely microalbuminuria, cystatin C, suggest that GPR‐120 may decline in tandem with advancing renal damage [21].

Our regression analyses highlight the interplay between GPR‐120 and dyslipidemia. Specifically, the positive coefficient for HDL‐C implies that individuals with better HDL profiles may preserve higher GPR‐120 levels, which in turn may mitigate renal stress. Meanwhile, elevated LDL‐C and triglycerides negatively correlated with GPR‐120, consistent with their established pro‐inflammatory and pro‐atherogenic roles [22]. These data indicate a potential “dual synergy” in which GPR‐120 and HDL‐C exert complementary protective effects, whereas high LDL‐C or triglycerides could disrupt that synergy, accelerating kidney injury. Although oxidized LDL was also elevated in nephropathy, its significance was attenuated when LDL‐C and triglycerides were included in the same model, suggesting possible overlapping mechanistic pathways [23-25].

Notably, eGFR emerged as a robust positive predictor of GPR‐120. One interpretation is that preserved renal filtration correlates with an overall healthier metabolic milieu, in which GPR‐120 is neither suppressed nor chronically consumed in inflammatory responses [26]. Conversely, once eGFR drops below threshold values, pathophysiological cascades (involving endothelial dysfunction, advanced glycation end products, and local cytokine release) may progressively reduce GPR‐120 [27,28]. Beyond statistical associations, biological plausibility supports a direct role for GPR-120 in renal protection. Activation of GPR-120 may reduce macrophage infiltration, suppress renal fibrosis via inhibition of transforming growth factor-β (TGF-β) signaling, and limit oxidative stress mechanisms implicated in both glomerular and tubular damage. Thus, declining GPR-120 may not only reflect renal injury but also contribute to its progression. Future studies are needed to determine whether restoring GPR-120 activity could ameliorate renal outcomes. Our study also highlights the potential utility of GPR-120 as a biomarker when used alongside conventional markers like cystatin C and microalbuminuria. Unlike these renal-specific indicators, GPR-120 may offer a broader perspective on systemic metabolic stress. Although its cost-effectiveness compared to current biomarkers was not evaluated, the per-test cost is relatively low, and its dual role in metabolic and renal health may justify its incorporation into risk-stratification algorithms pending further validation.

The findings of the present study highlight the potential value of measuring GPR-120 alongside conventional lipid and renal biomarkers in clinical practice. Validation of these results through prospective or interventional studies could enable earlier identification of diabetic patients at increased risk of renal dysfunction, particularly among those displaying concurrent abnormalities in lipid and GPR-120 profiles. Additionally, emerging evidence suggests that interventions aimed at modulating GPR-120 activity, such as supplementation with specific omega-3 fatty acids, may offer a protective effect against the progression of DN [29,30].

However, several limitations warrant consideration. First, the retrospective single-center design may limit generalizability and introduce selection bias. Second, nephropathy cases were not stratified by CKD stage due to sample size constraints, which limits our ability to evaluate stage-specific trends or include end-stage renal disease (ESRD) patients. Third, the influence of lipid-lowering drugs, antihypertensive agents, or glycemic control was not assessed due to the lack of stratification for medication use, BMI, duration of diabetes, or blood pressure. Fourth, the absence of multivariable modeling restricts causal interpretation. Fifth, the GPR-120 levels were not compared to glycemic parameters (HbA1C, FBS, PPBS), nor was diagnostic performance (sensitivity or specificity) evaluated against established markers.

We also did not include patients with non-diabetic kidney disease or those with comorbid liver disease, which could influence GPR-120 levels. Future prospective multicenter studies with diverse populations and longitudinal follow-up are essential to determine whether GPR-120 levels can predict the onset or progression of DN and how they compare in sensitivity and specificity to conventional biomarkers.

Our study contributes to the growing evidence that GPR‐120 may serve as a promising integrative biomarker linking metabolic and renal dysfunction in type 2 diabetes. While preliminary, these findings support further investigation into GPR‐120’s mechanistic role in kidney injury and its potential utility in clinical risk stratification.

## Conclusions

Serum GPR‐120 levels are significantly reduced amongst diabetic patients with nephropathy in comparison to ones without, and these decreased ranges correlate closely with adverse lipid profiles and declining renal parameters. Our results suggest that GPR‐120 can also function as an early risk factor indicator of metabolic‐renal dysfunction in type 2 diabetes. By providing greater insight into the link between renal pathophysiology and dyslipidemia, GPR-120 levels may enhance risk stratification and guide targeted interventions. Larger researches are needed to validate these findings and to discover novel therapeutic strategies aimed at restoring or augmenting GPR‐120.
